# Prevalence of pediatric sepsis in hospitalized children of Maiwand Teaching Hospital, Kabul, Afghanistan

**DOI:** 10.1186/s12887-023-04318-1

**Published:** 2023-10-16

**Authors:** Mohammad Sharif Sediqi, Abdulwali Wali, Mohammad Akbar Ibrahimi

**Affiliations:** Department of Pediatrics, Kabul University of Medical Sciences, P.O. Box 1003, Kabul, 2496300 Afghanistan

**Keywords:** Mortality, Prevalence, Pediatrics, Sepsis

## Abstract

**Background:**

Sepsis is a clinical syndrome associated with a systemic reaction to infection that is seen as a bacteremia with systemic symptoms. Sepsis is one of the most important problems in children and is associated with many deaths, so recognizing this disease and it’s causing factors and identifying the predisposing factors for it is of great importance. Globally, the prevalence and occurrences of sepsis and septic shock are increasing, while the incidence of deaths from them has decreased with the improvement of diagnostic and treatment facilities. According to a 2015 World Health Organization report, approximately 5.9 million children under 5 years old have lost their lives due to sepsis worldwide, the majority of which have occurred in developing countries.

**Methods:**

This study was conducted in the pediatric department of Maiwand Teaching Hospital (MTH) in 2020 as a descriptive cross-sectional study. All children who were admitted to the pediatric department of Maiwand Teaching Hospital during 2020 were included in the research. Among them, the prevalence of sepsis in children with respect to age and sex was studied. The study included children over the age of 28 days who were admitted to the Maiwand Teaching Hospital pediatrics department in 2020. However, in this study, patients have been categorized into five categories according to age: less than two months, two months to one year, one to three years, three to five years old, and older than five years old.

**Results:**

This study was conducted in the pediatric department of Maiwand Teaching Hospital in 2020 as a descriptive cross-sectional study, and it was found that the prevalence of sepsis in children who were admitted to the pediatric department at this year was 50.5%, including the highest prevalence in males (65.75%) and at the age of two months to one year (37.9%). In this study, it was found that the prevalence of sepsis was higher (88.46%) among urban children than children who were living in villages (11.53%). In this study, the mortality rate was 2.44% for patients admitted to Maiwand Teaching Hospital.

**Conclusions:**

In this study, it was found that the prevalence of sepsis was 50.5% in children admitted to the pediatrics department of Maiwand Teaching Hospital, of whom 67.75% were boys, 37.94% were aged two months to three years old, and it was more prevalent (88.46%) among children living in cities. The mortality rate was 2.44%.

## Background

Sepsis is the systemic inflammatory response syndrome, which is a common problem causing children to be hospitalized in intensive care all over the world [1]. Sepsis is a systemic response to Infection and is a clinical syndrome that is clinically associated with systemic symptoms [2]. Another study, conducted between 1997 and 2010 over 99,796 children in North America by Christoph P. Hornik MD, Prem Fort, MD, and published in 2012, found that 14,628 incidents (14.6%) were sepsis [2]. Other research conducted by A. Dawoda, AL. Umran, and K. Tawam-Danso with a cross-sectional study at the Royal Fahd Teaching Hospital in Saudi Arabia showed that out of a total of 1,291 hospitalized children, 61 children, 34 boys and 27 girls, had been suffering from sepsis [3, 4]. Another study conducted by Licia Maria from January 2016 to December 2016, a cross-sectional study in Ethiopia at the Clinic Porto Allergy Hospital, shared by 3,938 children, shows that 11.8% of children had sepsis [5, 6].

The diagnosis of sepsis is made by clinical manifestations and laboratory findings, with each child having tachycardia or bradycardia, tachypnea, fever or hypothermia, leukocytosis or leucopenia, a high ESR, positive C-reactive protein, and a positive blood culture, along with evidence of an infection source in one of the body’s systems. The existence of the above two symptoms, along with fever or hypothermia and changes in leukocytes, are among the essential criteria in the diagnosis of sepsis. In the world, the prevalence and occurrence of sepsis and septic shock are increasing, while the incidence of mortality has decreased with the improvement of diagnostic and treatment facilities. Epidemiological figures collected in developing countries are still not complete [7].

In 2015, the World Health Organization (WHO) recorded that approximately 5.9 million deaths of children fewer than 5 years of age were due to sepsis, and the majority of them occurred in developing countries because of infections including pneumonia, diarrhea, and malaria. These deaths occur when the severe form of the disease occurs; the word severe word indicates the state in which perfusion is reduced, acidosis occurs, and hypotension occurs. These conditions indicate severe septic states and are the cause of death [7]. In 2016, WHO conducted a systematic review study that showed the number of cases of sepsis in the world was 19 million (148 out of 100,000) and that 5% of deaths were caused by sepsis. In 2017, 49% of all sepsis incidents were recorded in the world, responsible for the deaths of 11% of children (189/100,000). In this report, it is mentioned that sepsis was caused by diarrhea and lower respiratory tract infections, and 41% of the incidents of sepsis (20 million children) were in children less than five years old. The overall mortality rate was 26.7%, and generally, the mortality rate among patients with sepsis treated in the ICU of hospitals was 42% [8]. Cross-sectional research conducted at Martomo Tri Utomo General Hospital in Indonesia from January 2010 to June 2010 found that out of a total of 337 hospitalized children, 36 (32%%), had sepsis, including 22 male children (61%), and 14 females (39%). In this study, we can see that there are more boys than girls who have sepsis [8]. Therefore, this problem is one of the causes of morbidity and death in children, and this study is carried out to study the prevalence of sepsis in order to prepare accurate figures of the situation and prevent its incidents. Over the past two decades, almost a definite majority (99%) of child deaths have been reported in developing countries, the majority of which are babies born at home, beyond the necessary care. Evidence suggests that children’s mortality can be prevented by simple methods such as awareness and care before, during, and after birth. Septicemia and meningitis in childhood are considered together due to their many similarities in pathology, epidemiology, pathophysiology, and clinical symptoms. The prevalence of septicemia among children in advanced countries is one in every 1,500 children with term babies and one in every 250 with preterm babies (about six times more than term babies) [9]. In research done by Zia. U. Rehman and his colleagues at Rehman Medical Complex hospital in Pakistan, which included 176 children and was published in 2021, the age of the children included in the research was 2.92 ± 1.32 years, including 61.9% boys and 38.1% girls [10].

Sepsis is a serious condition that affects the entire body. It is brought on by an infectious agent that enters the body of the sufferer and triggers the production of inflammatory mediators, which leads to SIRS. Sepsis is distinct from SIRS (Systemic Inflammatory Response Syndrome). It is a generalized inflammation that can happen as a result of trauma, infection, burns, pancreatitis, and numerous other illnesses [11, 12].

Rationale: By conducting this research, we can get documented results about sepsis outbreaks so that they can be used effectively in the future. In our country, due to many factors such as low laboratory capacity, poor reporting due to a lack of access to modern public health systems, and limited programs of surveillance, the prevalence of this pest is not known, and real figures cannot be provided throughout the country since having real numbers can draw more attention to such diseases that cause the most deaths and defects in children. Further, the necessary and effective measures were taken to treat and prevent it, so this research has been implemented.

## Methods

### Study design and participants

This study was conducted as a retrospective descriptive cross-sectional study. All patients who were admitted to the IPD section of the internal pediatric service of Maiwand Teaching Hospital (MTH) between January 1, 2020, and December 31, 2020, have been investigated. In the first stage, all patients who had been referred to IPD in 2020 were studied, and in the second stage, all sepsis patients were investigated. Those patients who did not have sufficient information in the register book or who did not have a legible and accurate record have not been investigated. MTH is a tertiary hospital with a 250-bed capacity and an annual admission of approximately 4000 patients. This study was approved by pediatrics department of MTH prior to commencement.

The study included pediatric patients younger than 15 years who had been admitted to the unit during the defined period and met the definition of sepsis.

Sepsis is a systemic inflammatory response syndrome associated with Infection or sepsis is a systemic response to infection. It is manifested by two or more of the SIRS (Systemic Inflammatory Response Syndrome) criteria as a consequence of documented or presumed infection. SIRS stands for Systemic Inflammatory Response Syndrome, and it involves symptoms such as fever, tachycardia, tachypnea, and leukocytosis [5]. Sepsis can lead to septic shock, which is a state of hypotension and hypo perfusion despite adequate fluid volume replacement. Sepsis and septic shock are associated with a loss of the redox balance, which can cause multiorgan failure and death [13].

Medical records were reviewed, and all patients were screened and evaluated for sepsis.

### Study definitions

Sepsis: Systemic inflammatory response syndrome with infection.

Age: In this study, children who were over 28 days old and under 15 years old were included. However, in this study, patients have been classified for ease of study into categories of less than two months, two months to one year, one to three years, three to five years old, and older than five years old.

Sex: Both sexes are included in the research.

### Data collection and analysis

Variables of interest included demographic data, underlying medical conditions, site of infection, and patient outcome. Patient data were collected from the computerized database and paper files.

The data were analyzed using descriptive statistics to evaluate the study population.

## Results

This study was conducted with the participation of 2675 patients who were hospitalized in the pediatric department of Maiwand Teaching Hospital in 2020, and the prevalence of sepsis was studied with them. The following results have been obtained:

During the study period, 2675 patients were admitted to the PICU, of whom 1301 were excluded because they did not meet the sepsis diagnosis criteria and 22 were excluded due to missing data. Ultimately, 1352 patients (50.5%) met the definition of sepsis and were enrolled. This research showed the prevalence of sepsis to be 50.5% among children [Table 1].

Meanwhile, it revealed that the prevalence of sepsis based on OPD data was 2.09% [Table 2].

The result showed a high prevalence of sepsis among children aged 2 months to 1 year [Table 3] and males [Table 4]. The distribution of sepsis according to place of residence was highest among children living in urban areas [Table 5]. And finally, the mortality rate was about 2.5% among these children [Table 6].

**Table 1:** Prevalence of patients with sepsis according to the total number of hospitalized patients

	Number	Percentage
Total number of patients hospitalized in the hospital in a year	2675	100
Sepsis	1352	50.5

**Table 2:** Prevalence of sepsis based on the total number of patients in OPD

	Number	Percentage
Total patients came to OPD in a year	64,500	100
Total sepsis patients	1352	2.09

**Table 3:** Age-wise prevalence of sepsis

Age groups	Number	%age
Less than two months	109	8.06
Two months to one year	513	37.94
One to three years	473	34.98
Three to five years	122	9.02
More than five years	135	9.98
Total	1352	100

**Table 4:** Prevalence of Sepsis According to Sex

Sex	Number	Percentage
Male	889	65.75
Female	463	34.24
Total	1352	100

**Table 5:** Distribution of Sepsis by Address

Address	Number	Percentage
Urban	1196	88.46
Rural	156	11.53
Total	1352	100

**Table 6:** Prevalence of Sepsis-related Mortality

Prognosis	Number	Percentage
Cured	1318	97.5
Died	34	2.5
Total	1352	100

## Discussion

Sepsis is the Systemic inflammatory response syndrome, which is a common problem causing children to be hospitalized in serious care all over the world [1]. Sepsis is a systemic response to Infection and is a clinical syndrome that is clinically associated with systemic symptoms [2]. Another study, conducted between 1997 and 2010 over 99,796 children in North America by Christoph P. Hornik MD, Prem Fort, MD, and published in 2012, found that 14,628 incidents (14.6%) were sepsis [2]. Other research conducted by A. Dawoda, AL. Umran, and K. Tawam-Danso with a cross-sectional study at the Royal Fahd Teaching Hospital in Saudi Arabia showed that out of a total of 1,291 hospitalized children, 61 children, 34 boys and 27 girls, had been suffering from sepsis [3, 4]. Another study conducted by Licia Maria from January 2016 to December 2016, a cross-sectional study in Ethiopia at the Clinic Porto Allergy Hospital, shared by 3,938 children, shows that 11.8% of children had sepsis [6].

This study was conducted in 2020 with the participation of 2675 patients who were admitted to the pediatric department of Maiwand Teaching Hospital by the descriptive cross-sectional method. In this study, it was found that the prevalence of sepsis was 50.5%, which is in accordance with the findings of other literature. Therefore, it can be concluded that the prevalence of sepsis in developed countries is lower, while it is more prevalent in developing countries. In a study conducted by Marjan Salahi and his colleagues in southern Iran at Yasuj University of Medical Sciences, 342 children were found to have the highest prevalence of sepsis at the age of less than three years (53.3%) [7]. In this study, the prevalence of sepsis based on gender was also studied, and it was found that the prevalence of sepsis in the male gender was 65.75% and in the female gender was 34.24%, which is the same as other researches. This study showed that boys were more likely to be affected by sepsis than girls [8]. As per the research conducted by Zia. U. Rehman and his colleagues at the Rahman Medical Complex Hospital in Pakistan with 176 children and published in 2021, it has been revealed that among them, 61.9% were boys and 38.1% were girls [10]. According to the results of the study, the result is almost identical to the above-mentioned research. In this study, the prevalence of sepsis in children according to age was studied, and it was found that two-month-olds to one-year-olds (37.94%) and one-year-olds to three-year-olds (34.98%) had the highest prevalence. In another study conducted by Hayun M. and his colleagues at private hospitals in Latin America entitled Prevalence and Outcome of Children Suffering from Sepsis, it shows that most of the children were 3.2–16.2 months old, which is 11.3 months old, i.e., the highest prevalence was median at 11 months of age, which is similar to our research. This study demonstrated a high pediatric sepsis mortality rate, comparable to that of other studies performed in this region [8, 9]. Moreover, in Egypt, the mortality rate of sepsis has been reported as 28.1 and 8.8% by Bekhit et al. and El-Mashad et al., respectively [8, 10]. As the cause of high mortality rate we can say there are a large number of factors that determine the outcome of children with sepsis in Afghanistan. We think some of these might be caused by delaying medical care, receiving therapy from local practitioners, using illogical antibiotic combinations, and routinely using steroids by a small number of unskilled local practitioners. Our study has some limitations including our study was cross-sectional in nature and assessed respondent perception of the obligations at a specific time and the results of this study may not be completely generalizable because the sample was restricted.

## Conclusions

In this study, it was found that the prevalence of sepsis was 50.5% in hospitalized patients; the prevalence of sepsis was high (67.75%) among boys, in the age range of two months to three years (37.94%), and among the children living in the cities (88.46%). The mortality rate was 2.44%.

The prevalence of sepsis in MTH can be compared to international data; however, the mortality rate remains high compared to developed countries. Implementation of a national database will help conduct large-scale studies to determine areas of weakness regarding the diagnosis of sepsis and therefore improve pediatric sepsis outcomes.

## Data Availability

The datasets used and/or analyzed during the current study are available from the corresponding author on reasonable request.

## Declarations

### Limitations

The study was limited by its retrospective design and the relatively small number of patients from a single center.
